# Supplementary material for: Criterion placement threatens the construct validity of neural measures of consciousness
Source: eLife. 2025 May 28;13:RP102335. doi: 10.7554/eLife.102335 (PMC12119085; doi:10.7554/eLife.102335)
Supplement: Supplementary file 1. — (a) Repeated measures ANOVA after post hoc sorting. In cases where Mauchly’s test of sphericity was violated, the Greenhouse–Geisser corrected values are provided below it on the second row. (b) Repeated measures ANOVA control (no post hoc sorting). In cases where Mauchly’s test of sphericity was violated, the Greenhouse–Geisser corrected values are provided below it on the second row. (c) Repeated measures ANOVA for Experiment 1 (detection), after post hoc sorting. There were no violations of Mauchly’s test of sphericity. (d) Repeated measures ANOVA for Experiment 2 (PAS), after post hoc sorting. In cases where Mauchly’s test of sphericity was violated, the Greenhouse–Geisser corrected values are provided below it on the second row. [file elife-102335-supp1.pdf]

Supplementary File 1a. Repeated Measures ANOVA after post hoc sorting). In cases where Mauchly’s test of sphericity was violated, the Greenhouse-Geisser corrected values are provided below it on the second row.

Within Subjects Effects

Cases	Sum of Squares	df	Mean Square	F	p	η^2_p
Criterion	0.025	1.000	0.025	20.984	4.455×10^{-5}	0.344
Criterion ✻ experiment	0.002	1.000	0.002	1.376	0.248	0.033
Residuals	0.047	40.000	0.001			
Latencies	0.213	2.000	0.106	33.719	2.395×10^{-11}	0.457
	0.213	1.787	0.119	33.719	2.222×10^{-10}	0.457
Latencies ✻ experiment	0.171	2.000	0.085	27.093	1.036×10^{-9}	0.404
	0.171	1.787	0.096	27.093	6.518×10^{-9}	0.404
Residuals	0.252	80.000	0.003			
	0.252	71.493	0.004			
Visibility	0.405	1.000	0.405	204.012	2.679×10^{-17}	0.836
Visibility ✻ experiment	0.073	1.000	0.073	36.888	3.725×10^{-7}	0.480
Residuals	0.079	40.000	0.002			
Criterion ✻ Latencies	0.009 [a]	2.000 [a]	0.004 [a]	4.442 [a]	0.015 [a]	0.100
	0.009	1.745	0.005	4.442	0.019	0.100
Criterion ✻ Latencies ✻ experiment	0.002 [a]	2.000 [a]	8.407×10^{-4} [a]	0.842 [a]	0.435 [a]	0.021
	0.002	1.745	9.635×10^{-4}	0.842	0.421	0.021
Residuals	0.080	80.000	9.990×10^{-4}			
	0.080	69.802	0.001			
Visibility ✻ Criterion	0.003	1.000	0.003	5.360	0.026	0.118
Visibility ✻ Criterion ✻ experiment	0.009	1.000	0.009	18.473	1.073×10^{-4}	0.316
Residuals	0.080	80.000	9.990×10^{-4}			
	0.080	69.802	0.001			
Visibility ✻ Latencies	0.194	2.000	0.097	71.794	1.398×10^{-18}	0.642
	0.194	1.829	0.106	71.794	3.372×10^{-17}	0.642
Visibility ✻ Latencies ✻ experiment	0.060	2.000	0.030	22.009	2.422×10^{-8}	0.355
	0.060	1.829	0.033	22.009	8.186×10^{-8}	0.355
Residuals	0.080	80.000	9.990×10^{-4}			
	0.080	69.802	0.001			
Visibility ✻ Criterion ✻ Latencies	0.003	2.000	0.001	2.561	0.084	0.060
	0.003	1.879	0.001	2.561	0.087	0.060
Visibility ✻ Criterion ✻ Latencies ✻ experiment	0.006	2.000	0.003	5.897	0.004	0.128
	0.006	1.879	0.003	5.897	0.005	0.128
Residuals	0.080	80.000	9.990×10^{-4}			
	0.080	69.802	0.001			

Note. Sphericity corrections not available for factors with 2 levels.

Note. Type III Sum of Squares

[a] Mauchly's test of sphericity indicates that the assumption of sphericity is violated ($p<.05$).

Between Subjects Effects

Cases	Sum of Squares	df	Mean Square	F	p	η^2_p
experiment	0.233	1	0.233	38.680	2.324×10^{-7}	0.492
Residuals	0.240	40	0.006			

Note. Type III Sum of Squares

Supplementary File 1b. Repeated Measures ANOVA control (no post hoc sorting). In cases where Mauchly's test of sphericity was violated, the Greenhouse-Geisser corrected values are provided below it on the second row.

Within Subjects Effects

Cases	Sum of Squares	df	Mean Square	F	p	η^2_p
Latencies	0.230 [a]	2.000 [a]	0.115 [a]	49.953 [a]	8.350×10^{-15} [a]	0.555
	0.230	1.699	0.135	49.953	6.320×10^{-13}	0.555
Latencies ✻ experiment	0.166 [a]	2.000 [a]	0.083 [a]	36.036 [a]	6.945×10^{-12} [a]	0.474
	0.166	1.699	0.098	36.036	1.953×10^{-10}	0.474
Residuals	0.184	80.000	0.002			
	0.184	67.954	0.003			
Criterion	2.869×10-4	1.000	2.869×10-4	1.164	0.287	0.028
Criterion ✻ experiment	3.225×10-4	1.000	3.225×10-4	1.309	0.259	0.032
Residuals	0.010	40.000	2.465×10-4			

Note. Sphericity corrections not available for factors with 2 levels.

Note. Type III Sum of Squares

[a] Mauchly's test of sphericity indicates that the assumption of sphericity is violated ($p<.05$).

Between Subjects Effects

Cases	Sum of Squares	df	Mean Square	F	p	η^2_p
experiment	0.212	1	0.212	28.446	4.070×10^{-6}	0.416
Residuals	0.297	40	0.007			

Note. Type III Sum of Squares

Supplementary File 1c. Repeated Measures ANOVA for experiment 1 (detection), after post hoc sorting. There were no violations of Mauchly's test of sphericity.

Within Subjects Effects

Cases	Sum of Squares	df	Mean Square	F	p	η^2_p
Criterion	0.016	1	0.016	13.890	0.002	0.481
Residuals	0.017	15	0.001			
Latencies	0.289	2	0.144	37.129	7.677×10^{-9}	0.712
Residuals	0.117	30	0.004			
Visibility	0.332	1	0.332	81.376	1.904×10^{-7}	0.844
Residuals	0.061	15	0.004			
Criterion ✻ Latencies	0.007	2	0.004	3.477	0.044	0.188
Residuals	0.032	30	0.001			
Visibility ✻ Criterion	0.008	1	0.008	11.056	0.005	0.424
Residuals	0.032	30	0.001			
Visibility ✻ Latencies	0.175	2	0.088	30.405	6.093×10^{-8}	0.670
Residuals	0.032	30	0.001			
Visibility ✻ Criterion ✻ Latencies	0.007	2	0.003	3.842	0.033	0.204
Residuals	0.032	30	0.001			

Note. Type III Sum of Squares

Supplementary File 1d. Repeated Measures ANOVA for experiment 2 (PAS), after post hoc sorting. In cases where Mauchly's test of sphericity was violated, the Greenhouse-Geisser corrected values are provided below it on the second row.

Within Subjects Effects

Cases	Sum of Squares	df	Mean Square	F	p	η^2_p
Criterion	0.025	1.000	0.025	10.102	0.004	0.288
Residuals	0.062	25.000	0.002			
Latencies	0.162	2.000	0.081	13.256	2.404×10^{-5}	0.347
	0.162	1.891	0.085	13.256	3.679×10^{-5}	0.347
Residuals	0.305	50.000	0.006			
	0.305	47.277	0.006			
Visibility	0.175 [a]	3.000 [a]	0.058 [a]	67.287 [a]	3.216×10^{-21} [a]	0.729
	0.175	2.369	0.074	67.287	3.078×10^{-17}	0.729
Residuals	0.065	75.000	8.677×10^{-4}			
	0.065	59.222	0.001			
Criterion ✻ Latencies	0.006	2.000	0.003	1.769	0.181	0.066
	0.006	1.655	0.004	1.769	0.188	0.066
Residuals	0.088	50.000	0.002			
	0.088	41.371	0.002			
Visibility ✻ Criterion	0.004 [a]	3.000 [a]	0.001 [a]	2.863 [a]	0.042 [a]	0.103
	0.004	2.292	0.002	2.863	0.058	0.103
Residuals	0.088	50.000	0.002			
	0.088	41.371	0.002			
Visibility ✻ Latencies	0.108 [a]	6.000 [a]	0.018 [a]	26.049 [a]	4.358×10^{-21} [a]	0.510
	0.108	4.210	0.026	26.049	1.816×10^{-15}	0.510
Residuals	0.088	50.000	0.002			
	0.088	41.371	0.002			
Visibility ✻ Criterion ✻ Latencies	0.005 [a]	6.000 [a]	7.515×10^{-4} [a]	1.055 [a]	0.392 [a]	0.040
	0.005	4.045	0.001	1.055	0.383	0.040
Residuals	0.088	50.000	0.002			
	0.088	41.371	0.002			

Note. Sphericity corrections not available for factors with 2 levels.
Note. Type III Sum of Squares
[a] Mauchly's test of sphericity indicates that the assumption of sphericity is violated (p<.05).
